# The South African Rugby Injury and Illness Surveillance and Prevention Project (SARIISPP): SARU Girls’ Youth Week Injury Surveillance Report, 2022

**DOI:** 10.17159/2078-516X/2024/v36i1a18554

**Published:** 2024-05-15

## Executive Summary

As part of the South African Rugby (SARU) Injury and Illness Surveillance and Prevention Project (SARIISPP), SARU records and investigates injury data from their annual SARU Youth Week tournaments. The BokSmart National Rugby Safety Programme has consistently gathered and analysed these data since 2011 for the SARU Boy’s Youth Week tournaments. In 2015, the project expanded to the SARU Girls’ Youth Week data collection.

This report focuses on the two provincial SARU Girls’ Youth Week tournaments in 2022: Girls Under 16 Week (Gu16W) and Girls Under 18 Week (Gu18W). Each tournament consisted of 32 teams and 32 matches were played. Comparisons between the two SARU Girls’ Youth Week tournaments (Gu16W vs. Gu18W) are made for the years 2015 to 2022. It must be noted that no Gu16W tournament was held in 2017. Additionally, no Gu16W and Gu18W tournaments were held in 2020 and 2021 due to the COVID-19 pandemic. The 2022 tournament format was amended so that each age group only played twice during the week. This was done to allow for a rest day to be inserted between each allocated playing day.

Each medical facility at the SARU Youth Week tournaments has a designated researcher(s) on site, who together with the tournament medical doctors, records the tournament injury data daily. This analysis of the data investigates injury profiles and patterns for the SARU Girls’ Youth Week tournaments (u16 and u18) over time. Furthermore, the analysis compares the profiles of injured players at each tournament. Potential areas of concern such as changes in the game, tournament structure or medical support services, can be identified while investigating these patterns. In turn, injury-specific interventions can be developed and implemented where the evidence supports such a need.

In 2022, there were 82 time-loss injuries for both tournaments (GuU16W and Gu18W) combined. This equated to an average of 79 (62 to 96) injuries per 1000 player hours [data are expressed as mean (95% confidence intervals) injuries per 1000 player hours]. The Time-Loss injury incidence for the Gu16W and Gu18W tournaments were 81 (56 – 107) injuries per 1000 player hours and 77 (54 – 100) injuries per 1000 player hours, respectively. Combining the injury incidence data collected over the five years, there was no significant difference between the two age groups. Injury Rate Ratio (IRR) was used in this report to determine the difference between injury incidences in 2019 and 2022. The injury incidences reported in both tournaments in 2022 were about 4-fold higher than the incidences recorded before COVID-19 in 2019 (Gu16W IRR = 3.7 (2.3 to 5.9), Gu18W IRR = 4.1 (2.5 to 6.6)).

In 2022, *Tackling* was the most frequent injury-causing event, followed by *Being Tackled*, and then *Open Play*. *Tackling front-on (regulation)* and *Tackling side-on (regulation)* were the most frequent injury-causing mechanisms involved in the Tackler injuries. *Being Tackled front-on (regulation)* and *Being Tackled side-on (regulation)* were the most frequent injury-causing mechanisms involved in Ball Carrier injuries. Lastly, *Collision* was the most frequent injury-causing mechanism in *Open Play*.

The most common injury type for the combined tournaments was Central Nervous System injuries, where Gu16W recorded the highest incidence. Head and Neck were the most common injury locations in 2022, accounting for 48% of the injuries, with the highest number occurring in the Gu16W. Players who started the match sustained more injuries compared to players who joined the match as substitutions. Locks and Loose forwards were the player positions with the highest normalised injury incidence per player per position across both tournaments.

As expected, the injury incidence of *‘New’* injuries was higher than subsequent ‘*Recurrent’* injuries. Most ‘*New*’ injuries were muscle injuries, while most *‘Recurrent’* injuries were joint/ligament injuries.

Thirty (30) concussions were recorded in 2022, which is sizably higher than the number of concussions recorded in 2019. Furthermore, the act of *Tackling* contributed to 40% of the events causing concussions. The Concussion rate increased 5-fold for Gu16W and 2-fold for Gu18W compared to the 2019 tournament, prior to the COVID-19 pandemic period; (IRR = 5.1 (2.2 to 11.2) for Gu16W and IRR = 2.1 (1.0 to 4.2) for Gu18W).

This report recommends keeping the current competition format unchanged until the Girls Youth Game at schools and youth clubs becomes more established. Furthermore, an increased emphasis should be on making young female rugby players more confident and proficient in contact situations. Progressive and tailored training of tackling and ball-carrying techniques in junior South African female rugby players must be prioritised to develop the basic contact-safety skills, techniques, and required levels of proficiency to minimise concussions and rugby injuries in general. Introducing the “Preparation for Contact” and “Contact Confident” programmes, freely available on the World Rugby education platform, may assist with the training and contact preparation of junior South African female rugby players.

**Figure f24-2078-516x-36-v36i1a18554:**
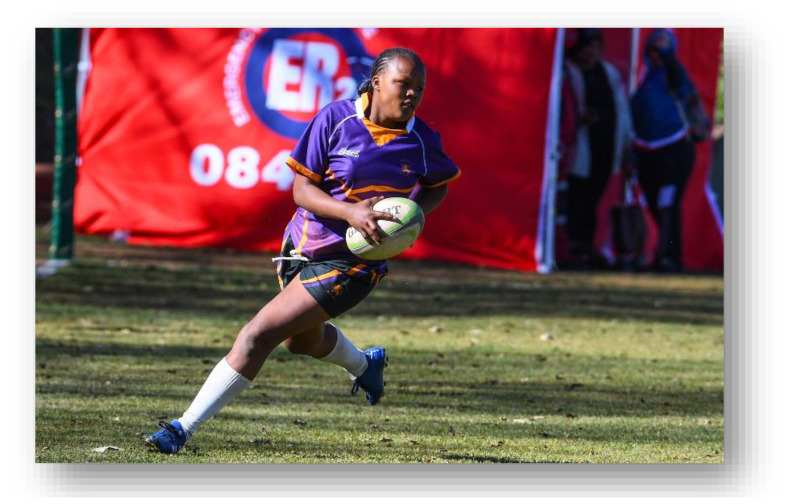

[Fig f24-2078-516x-36-v36i1a18554][Fig f25-2078-516x-36-v36i1a18554][Fig f26-2078-516x-36-v36i1a18554][Fig f27-2078-516x-36-v36i1a18554][Fig f28-2078-516x-36-v36i1a18554][Fig f29-2078-516x-36-v36i1a18554][Fig f30-2078-516x-36-v36i1a18554][Fig f31-2078-516x-36-v36i1a18554][Fig f32-2078-516x-36-v36i1a18554][Fig f33-2078-516x-36-v36i1a18554][Fig f34-2078-516x-36-v36i1a18554][Fig f35-2078-516x-36-v36i1a18554][Fig f36-2078-516x-36-v36i1a18554][Fig f37-2078-516x-36-v36i1a18554][Fig f38-2078-516x-36-v36i1a18554][Fig f39-2078-516x-36-v36i1a18554]

**Figure f25-2078-516x-36-v36i1a18554:**
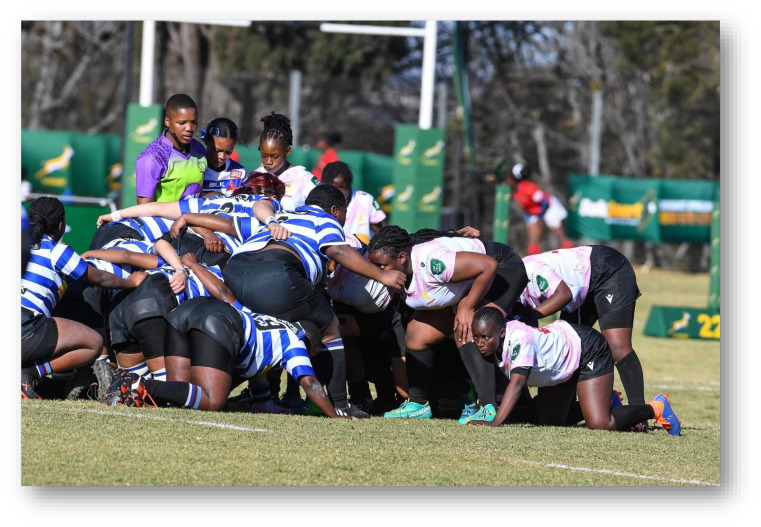

## Definitions

All definitions are originally based on the 2007 consensus statement for injury reporting in rugby union (1) and have since been realigned with the latest International Olympic Committee (IOC) consensus statement for methods of recording and reporting epidemiological data on injury and illness in sport (2).

### MEDICAL ATTENTION INJURY

All injuries seen by the tournament medical doctor or medical support staff were classified as Medical Attention injuries. These injuries are defined by the 2007 statement as an “*injury that results in a player receiving medical attention”* (1), and by the more recent IOC statement as *“a health problem that results in an athlete receiving medical attention”* (2).

### TIME-LOSS INJURY

Medical Attention injuries were further categorised as Time-Loss injuries, where appropriate, and defined by the 2007 statement as, “*an injury that results in a player being unable to take a full part in future rugby training or match play*” (1). The IOC definition is, *“a health problem that results in a player being unable to complete the current or future training session or competition”* (2). For clarity, this means an injury sustained by a rugby union player during a match or training session that prevented or would have prevented the player from taking full part in all rugby training activities and/or match play for more than 1 day following the day of injury, irrespective of whether match or training sessions were scheduled (3).

### INJURY RATE

This report defines an injury rate as the number of injuries expressed per 1000 player exposure hours. This method of expressing the rate of injury has been used in previous Youth Week reports and other international literature. As a result, it is easy to make comparisons. Moreover, the injury rate is expressed as a mean with 95% confidence intervals. A 95% confidence interval around a mean value indicates a 95% chance (i.e., very high chance) that the true value falls within this range. In this report, we present the 95% confidence intervals assuming a normal distribution of the data and use the approach of examining the overlap of the confidence intervals to determine whether the injury incidences are significantly different; if the range of confidence interval values of two comparisons does not overlap, there is a strong chance (95%) that their injury rates are different from each other. We have opted for this method because it is easy to use, conservative and less likely to produce false positive results (4).

### NEW, SUBSEQUENT AND RECURRENT INJURIES

In the 2022 Girls’ Youth Weeks, a ‘*New Injury’* was defined as when a player sustained her first injury. Any injury the *same* player sustained after this initial injury was defined as a *‘Subsequent Injury’.*

According to the IOC statement, any subsequent injury to the same site and of the same type is referred to as a ‘*Recurrence’* if the index injury was fully recovered before reinjury, and as an *‘Exacerbation’* if the index injury was not yet fully recovered (2).

To provide more detail on the subsequent injuries for practitioners, we have further categorised the subsequent injuries in this report into one of four groups based on the Orchard Sports Injury and Illness Classification System (OSIICS) classification diagnosis:

- Different site - Different type
- Different site - Same type
- Same site - Different type
- Same site - Same type

According to the 2007 Consensus Statement for rugby, any subsequent injury classified as ‘Same site - Same type’ was a *‘Recurrent injury’* (1).

**Figure f26-2078-516x-36-v36i1a18554:**
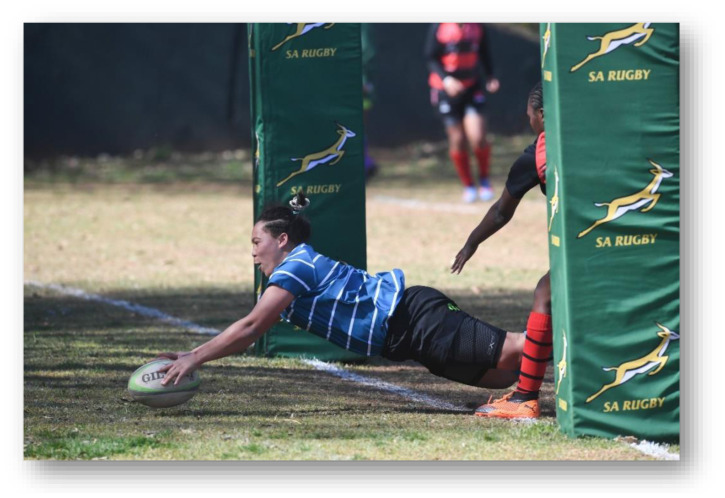

## Key Findings

### Injury Incidence

**Figure 1:**
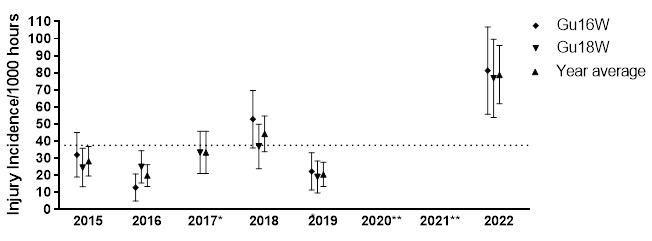
Injury incidence/1000 player hours and 95% confidence intervals of injuries for the SARU Girls’ Youth Week tournaments from 2015 – 2022. The dotted line reflects the average incidence for all tournaments over all the included years. ^*^No Gu16W tournament was held in 2017. ^**^No Gu16W and Gu18W tournaments were held in 2020 and 2021 due to the COVID-19 pandemic.

In 2022, thirty-two teams participated in the SARU Girls’ Youth Week tournaments (Gu16W = 16 teams, Gu18W = 16 teams). A total of 121 Medical Attention injuries were recorded during the tournaments. Sixty-eight percent of these (n = 82) were Time-Loss injuries. The combined tournaments’ injury incidence and 95% confidence intervals for all Medical Attention injuries was 116 (96 to 137) injuries/1000 player hours, and for Time-Loss injuries was 79 (62 to 96) injuries/1000 player hours. No significant difference was found between the two SARU Girls’ Youth Week tournaments in 2022 (Table 1). Table 2 represents the Medical Attention and Time-Loss injuries per match and per hour of match play across both tournaments. On average, there were approximately 5 time-loss injuries for every two matches played. Figure 1 shows the pattern of Injury incidence/1000 player hours and 95% confidence intervals of Time-Loss injuries for each tournament (2015 to 2022). It must be noted that the Gu16W tournament in 2017 did not take place and both Gu16W and Gu18W did not occur in 2020 and 2021 due to the COVID-19 pandemic (Figure 1). Injury Rate Ratio (IRR) was used in this report to determine the extent of the difference in injury incidence between 2019 (prior to COVID-19) and 2022 (post COVID-19). Injury incidence/1000 player hours and 95% confidence intervals of Time-Loss injuries reported in both tournaments in 2022 were significantly higher than the incidences recorded before the COVID-19 pandemic: about 4-fold higher than 2019’s injury incidence rates (Gu16W IRR = 3.7 (2.3 to 5.9), Gu18W IRR = 4.1 (2.5 to 6.6)).

**Figure 2:**
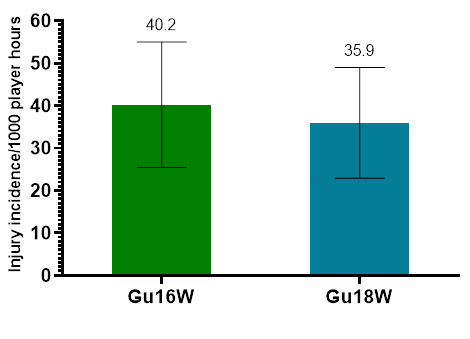
Average injury incidence/1000 player hours and 95% confidence intervals for the SARU Girls’ U16 (Gu16W) and U18 (Gu18W) Youth Week tournaments from 2015 – 2022. The number above each bar represents the average injury incidence/1000 player hours over the time-period of data collection.

Only Time-Loss injuries were analysed further in this report (n = 82). Figure 2 illustrates a slightly lower combined injury incidence in Gu18W (2015 to 2022) compared to Gu16W. In general, there were no significant differences in the injury incidence rates between these two tournaments.

**Figure f27-2078-516x-36-v36i1a18554:**
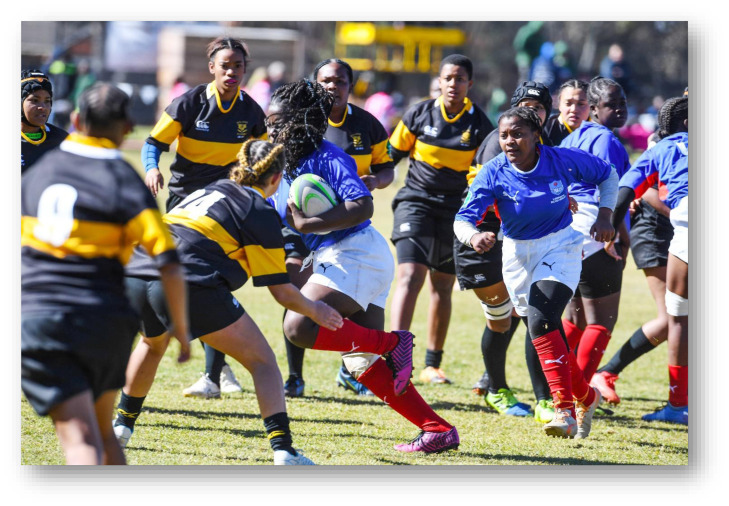

**Figure f28-2078-516x-36-v36i1a18554:**
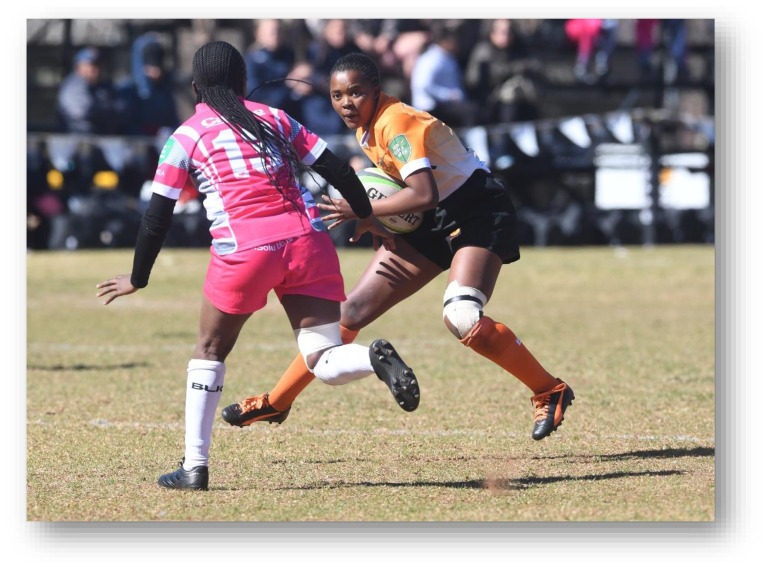

### Injury Incidence Trends

#### Girls U16 Week (Gu16W)

**Figure 3:**
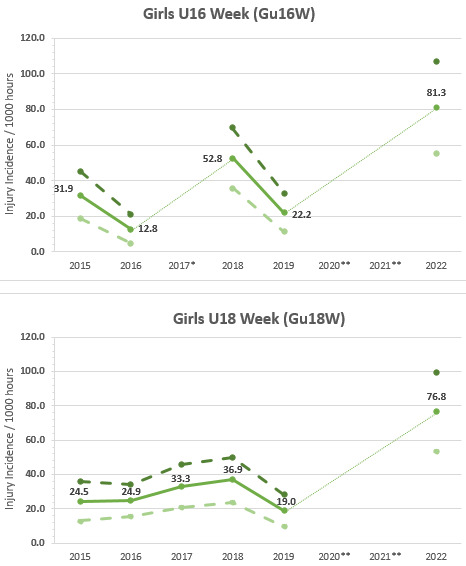
Time-Loss Injury incidence for each SARU Girls’ Youth Week tournament per year from 2015 – 2022, including the upper and lower 95% Confidence Intervals (95%CI). ^*^No Gu16W tournament was held in 2017. ^**^No Gu16W and Gu18W tournaments were held in 2020 and 2021 due to the COVID-19 pandemic.

The Gu16W tournament was not held in 2017, 2020 and 2021; therefore, the trendline could not be calculated accurately and has been excluded. The highest injury incidence to date was recorded in 2022, with a large upward spike in injuries from 2019 (Figure 3).

#### Girls U18 Week (Gu18W)

The Gu18W tournament was not held in 2020 and 2021; therefore, the trendline could not be calculated accurately and has also been excluded. After a slight decrease in 2019, as with the Gu16W, there was a significant increase in injury incidence in 2022 at the Gu18W (Figure 3). The injury incidence in 2019 was the lowest in the 5 years of study and the increase in 2022 was the highest (Figure 3).

**Figure f29-2078-516x-36-v36i1a18554:**
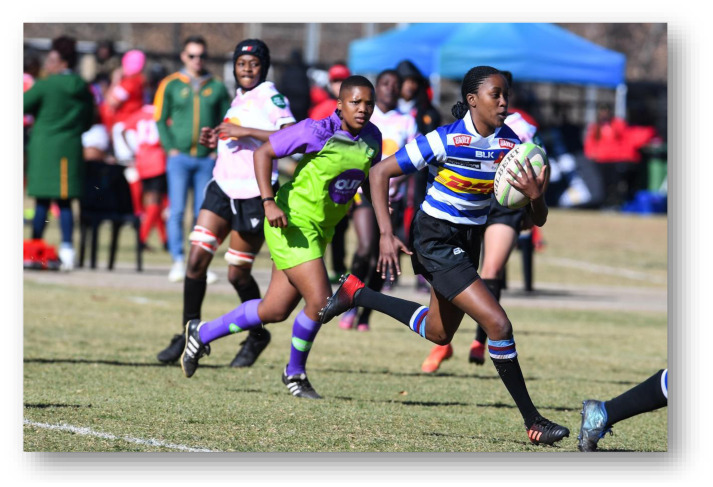

## Injury Event

In 2022, the *Tackler* role was the event associated with the most injuries throughout the tournaments (34%), followed by the *Ball Carrier* role (26%) and then *Open Play* (16%). *Tacklers* recorded 27 (17 to 37) injuries/1000 player hours, while *Ball Carrier* injuries had an injury incidence of 20 (12 to 29) injuries/1000 player hours, with injuries in *Open Play* at 13 (6 to 19) injuries/1000 player hours. In the Gu18W tournament, *Tackler* injuries dominated, while in the Gu16W, *Tacklers* and *Ball Carriers* shared the most injury-causing event. (Table 3).

**Figure 4:**
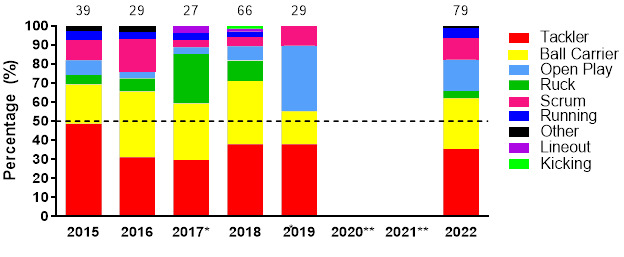
Most common injury-causing events in the SARU Girls’ Youth Week tournaments from 2015 – 2022. (The number above each bar represents the total number of injuries for that year). Missing 2022 data = 3 cases. ^*^No Gu16W tournament was held in 2017. ^**^No Gu16W and Gu18W tournaments were held in 2020 and 2021 due to the COVID-19 pandemic.

Figure 4 displays the grouped proportional breakdown of injuries between 2015 and 2022, resulting from the different injury-causing events. The proportions of *Tackler* and *Ball Carrier* injuries fluctuated throughout the years but have remained the most prominent injury-causing events. The Tackle event (both *Tackler* and *Ball carrier* combined) ranged between 55% – 71% (averaging 64%) of all injuries per year in the Girls’ Youth Weeks (both Gu16W and Gu18W) during this period. There was an increase in injuries to the *Ball Carrier* and a decrease in injuries occurring in *Open Play* between 2019 and 2022 (no tournaments were played in 2020 and 2021).

**Figure f30-2078-516x-36-v36i1a18554:**
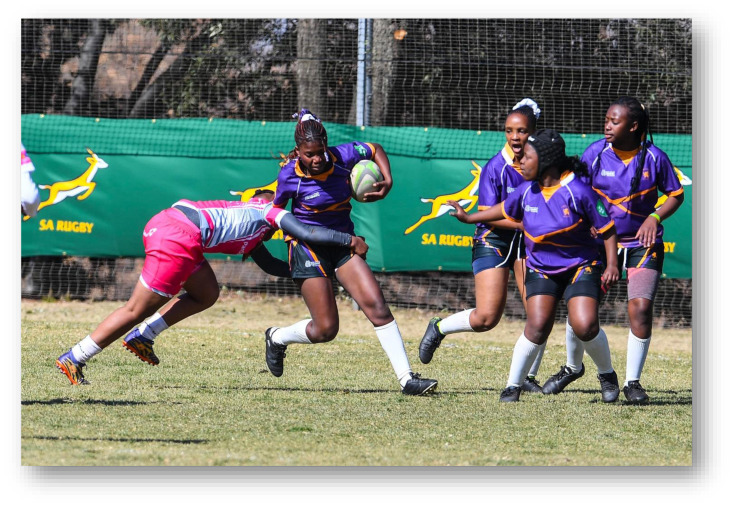

**Figure 5:**
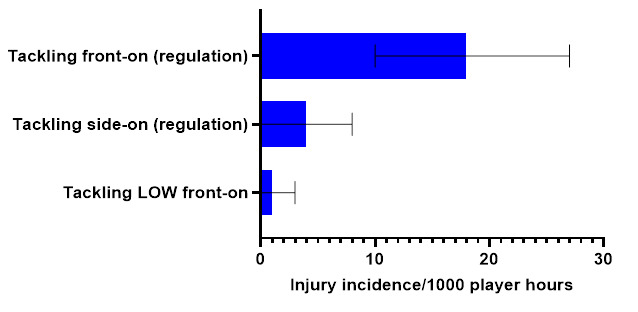
Injury incidence and 95% confidence intervals/1000 player hours of Tackler-related injury mechanisms at the 2022 SARU Girls’ Youth Week Tournaments. Missing 2022 data = 3 cases.

Ranked from highest to lowest, in 2022, *Tackling front-on (regulation)*, *Tackling side-on (regulation)*, and *Tackling LOW front-on* were the mechanisms that accounted for injuries to *Tacklers*. *Tackling front-on (regulation)* accounted for 68% of *Tackler injuries*, with 18 (10 to 27) injuries/1000 player hours (Figure 5).

**Figure 6:**
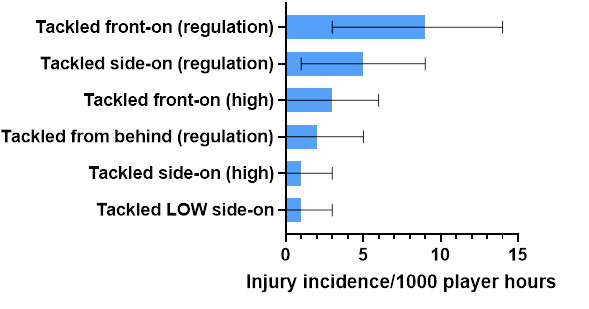
Injury incidence and 95% confidence intervals/1000 player hours of Ball Carrier-related injury mechanisms at the 2022 SARU Girls’ Youth Week Tournaments. Missing 2022 data = 0 cases.

Ranking the mechanisms of injury to *Ball Carrier*s from highest to lowest contributors, in 2022, *Tackled front-on (regulation)* was the highest, followed by *Tackled side-on (regulation)*, *Tackled front-on (high), Tackled from behind (regulation)*, with the lowest being shared between *Tackled side-on (high)* and *Tackled LOW side on. Tackled front on (regulation)* accounted for 43% of these injuries, with an injury incidence of 9 (3 to 14) injuries/1000 player hours (Figure 6).

**Figure 7:**
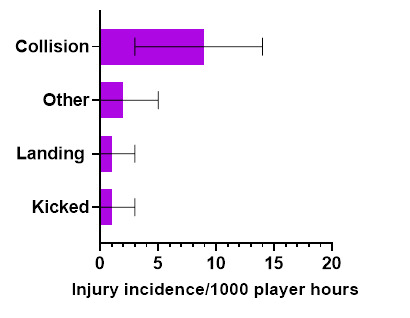
Injury incidence and 95% confidence intervals/1000 player hours for Open Play-related injury mechanisms at the 2022 SARU Girls’ Youth Week Tournaments. Missing 2022 data = 0 cases.

*Collision in Open Play* accounted for the highest proportion of Open Play injuries (69%), with 9 (3 to 14) injuries/1000 player hours (Figure 7).

**Figure f31-2078-516x-36-v36i1a18554:**
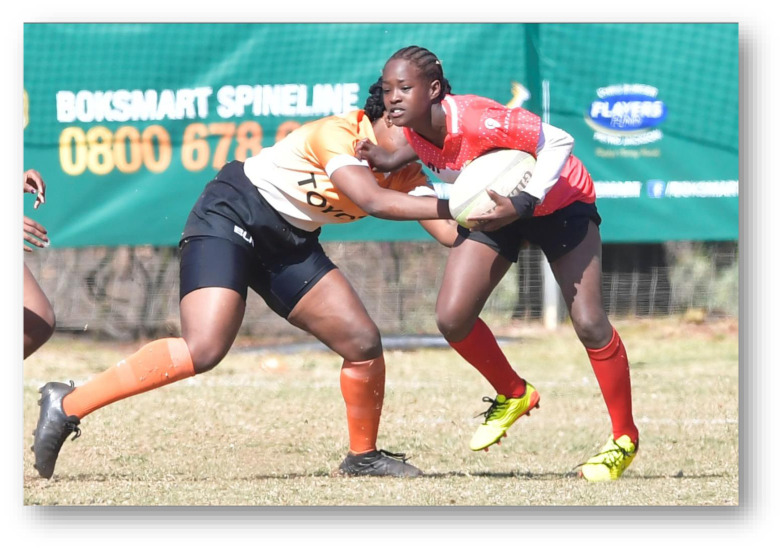

## Injury Type

In 2022, CNS (Central Nervous System) injuries were the most common injury type (Table 4). There were significantly more CNS injuries than Joint/Ligament injuries at the Gu18W tournament and in the combined data across both tournaments. There were no significant differences between injury types at the Gu16W tournament.

**Figure 8:**
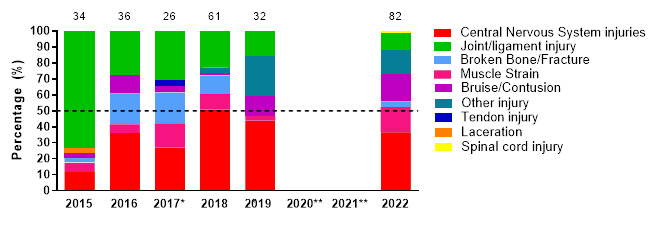
Most common injury types in the SARU Girls’ Youth Week tournaments from 2015 – 2022. (The number above each bar represents the total number of injuries for that year). *No Gu16W tournament was held in 2017. **No Gu16W and Gu18W tournaments were held in 2020 and 2021 due to the COVID-19 pandemic. Missing 2022 data = 0 cases.

Figure 8 displays the most common injury types in proportionate contributions per year from 2015 to 2022. The proportionate percentage of CNS and joint/ligament injuries decreased from 2019 to 2022 but remained the two most common injury types in the SARU Girls’ Youth Week tournaments over the years. It is worth noting that since the first tournament data collected in 2015, there has been a slow but consistent reduction in the proportionate contribution of Joint/Ligament injuries. It will be interesting to observe if this reduction continues in the coming years. CNS contributed to 44% and 37% of all injuries in 2019 and 2022, respectively. Muscle strain, Bruise/Contusion, and Broken Bone/Fracture Injuries increased in 2022.

## Body Location

Injuries were grouped according to the four main body location groups (*Head and Neck; Trunk; Upper Body; Lower Body*) across both tournaments. In 2022, the most common injured body location was the *Head and Neck* (48%), with 56% of these injuries occurring at the Gu16W tournament. *Head and Neck* injuries accounted for an injury incidence of 38 (26 to 49) injuries/1000 player hours (Table 5). The Gu16W had the highest *Head and Neck* injury incidence recorded at 46 (27 to 65) injuries/1000 player hours, but this was not significantly different from the Gu18W tournament. Following *Head and Neck* injuries, *Lower Body* injuries were the second most common. *Lower Body* injuries accounted for an injury incidence of 28 (18 to 38) injuries/1000 player hours. Gu18W had the highest *Lower Body* injury incidence of 30 (16 to 45) injuries/1000 player hours which was also not significantly different from the Gu16W tournament.

**Figure f32-2078-516x-36-v36i1a18554:**
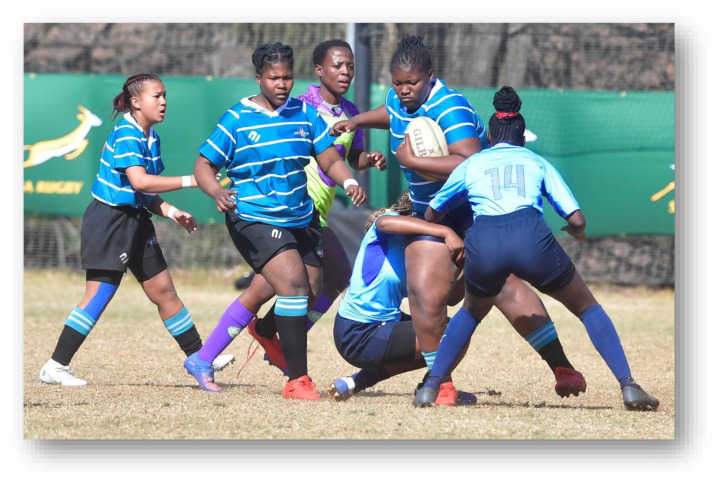

The IOC Consensus statement’s recommended categories of Tissue and Pathology injury data are presented in Table 6 for the 2022 SARU Girls’ Youth Week tournaments (2).

## Match Status

**Figure 9:**
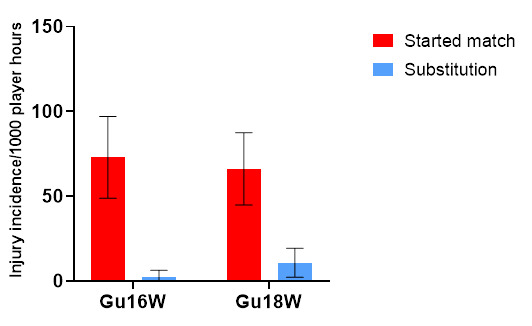
Injury incidence and 95% confidence intervals/1000 exposure hours of players who started the match and those who came on as substitutes in the 2022 SARU Girls’ Youth Week tournaments. Missing 2022 data = 3 cases.

In 2022, there were significantly more injuries to players who started the match (91%) compared to players who joined the match as substitutions (9%) (Figure 9). There were no other significant differences in injury rates between tournaments or in players who came on as substitutes (Table 7).

**Figure f33-2078-516x-36-v36i1a18554:**
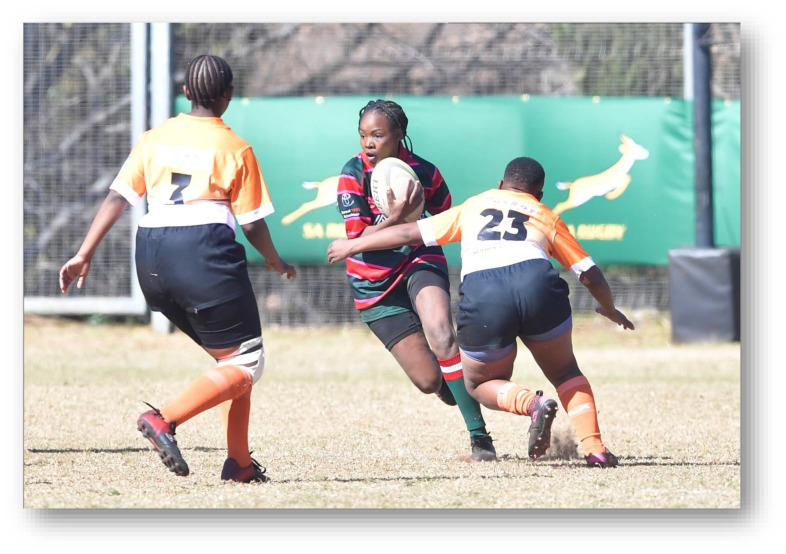

## New vs recurrent

The injury incidence of *‘New’* injuries in 2022 was 70 (54 to 86) injuries/1000 player hours; significantly higher than ‘*Recurrent’* injuries which had an injury incidence of 9 (3 to 14) injuries/1000 player hours.

**Figure 10:**
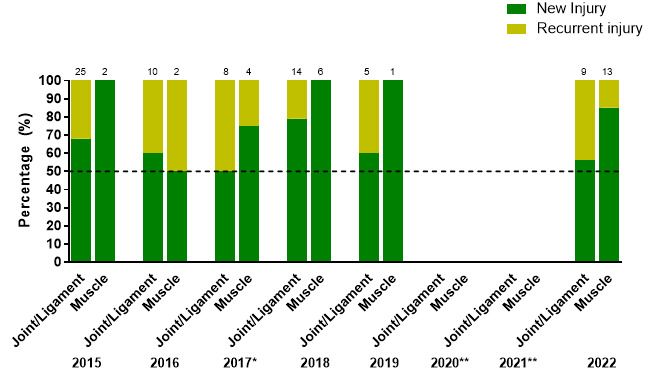
Proportion of New and Recurrent joint/ligament and muscle injuries in the SARU Girls’ Youth Week tournaments from 2015 – 2022. (The number above each bar represents the total number of injuries for that year). *No Gu16W tournament was held in 2017. **No Gu16W and Gu18W tournaments were held in 2020 and 2021 due to the COVID-19 pandemic.

Figure 10 illustrates the proportion of *‘New’* and *‘Recurrent’* joint/ligament and muscle injuries across the years (2015–2022).

The proportion of *‘New’* joint/ligament injuries is similar in 2019 (60%) and 2022 (56%). *‘New’* muscle injuries decreased proportionately from 2019 (100%) to 2022 (85%).

‘*Recurrent’* joint/ligament injuries were similar in 2019 (40%) to 2022 (44%) and ‘*Recurrent’* muscle injuries increased from 2019 (0%) to 2022 (15%).

The injury numbers in 2019 were however very low and therefore these changes need to be interpreted with caution.

## Game Quarter

**Figure 11:**
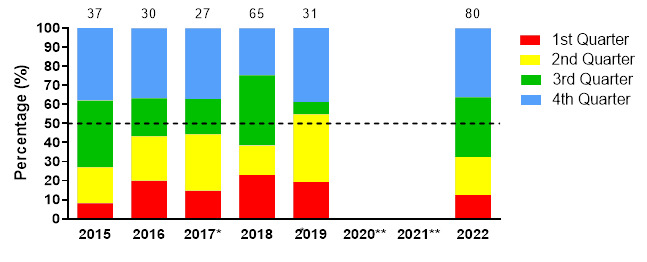
Proportion of injuries occurring in each game quarter in the SARU Girls’ Youth Week tournaments from 2015 – 2022. (The number above each bar represents the total number of injuries for that year). Missing 2022 data = 1 case. *No Gu16W tournament was held in 2017. **No Gu16W and Gu18W tournaments were held in 2020 and 2021 due to the COVID-19 pandemic.

In 2022, most injuries occurred in the 4^th^ quarter (36%) followed by the 3^rd^ quarter (31%) with an injury incidence of 28 (18 to 38) injuries/1000 player hours and 24 (15 to 34) injuries/1000 player hours respectively. A significant increase in injuries occurred in the 3^rd^ match quarter from 2019 to 2022 (Figure 11).

**Figure f34-2078-516x-36-v36i1a18554:**
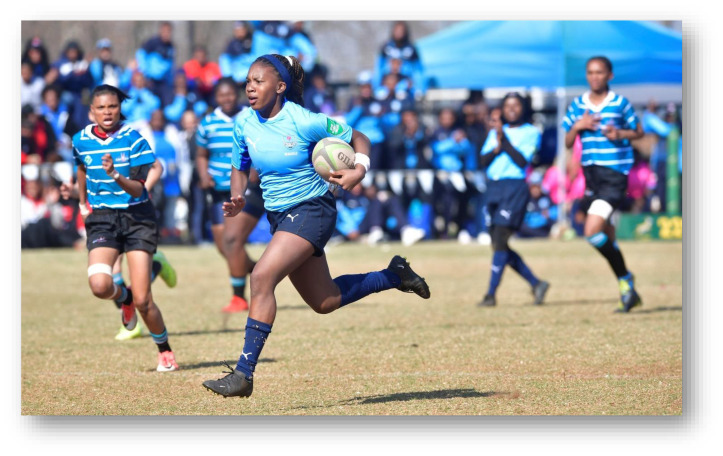

## Player positions

**Figure 12:**
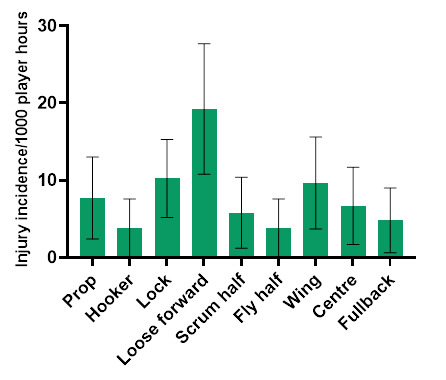
Absolute injury incidence and 95% confidence intervals/1000 player hours per positional grouping in the combined 2022 SARU Girls’ Youth Week Tournaments. Missing 2022 data = 2 cases.

Loose forwards and locks had an *absolute* injury incidence of 19 (11 to 28) injuries/1000 player hours and 15 (8 to 23) injuries/1000 player hours, respectively (Figure 12). *Absolute* incidence refers to the incidence of injury in a player’s positional grouping, e.g., wings, without normalising for the number of players on the field playing in that positional grouping, e.g., there are two wings per team on the field. In 2022, the loose forward and lock positions had the highest *absolute* injury incidence rates across the SARU Girls’ Youth Week tournaments.

The number of injuries was also *normalised* to the number of players on the field in a positional grouping. For example: Props = total number of injuries divided by 2, Locks = total number of injuries divided by 2, Loose forwards = total number of injuries divided by 3.

**Figure 13:**
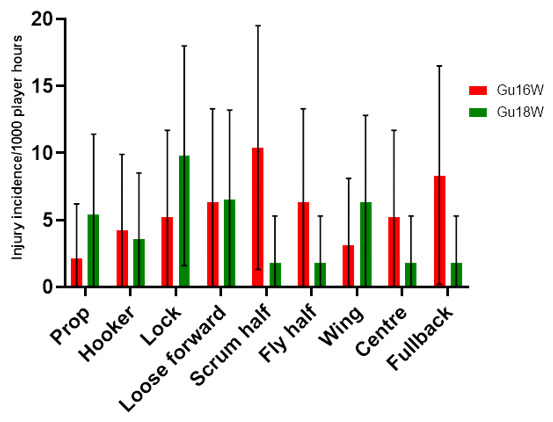
Normalised injury incidence and 95% confidence intervals/1000 player hours per player per position in the two 2022 SARU Girls’ Youth Week Tournaments. Missing 2022 data = 2 cases.

**Figure 14:**
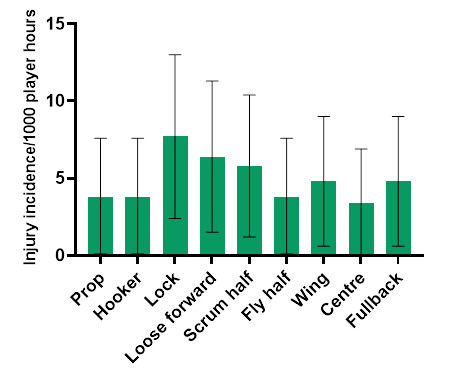
Normalised injury incidence and 95% confidence intervals/1000 player hours per player per position, across the combined 2022 SARU Girls’ Youth Week Tournaments. Missing 2022 data = 2 cases.

Figure 13 shows the *normalised* injury incidence per player per position across the two tournaments. In Gu16W, Scrum halves and Fullbacks stood out, and in the Gu18W, Locks, Loose forwards and Wings were the standout positions. Figure 14 shows the combined *normalised* positional injury rates across both tournaments. In 2022, when combining the data, the lock and loose forward positions had the highest *normalised* injury incidence rates across both tournaments. Locks, when normalised per player, had an injury incidence of 8 (2 to 13) injuries/1000 player hours and loose forwards had an injury incidence of 6 (2 to 11) injuries/1000 player hours (Figure 14).

## Concussion

In 2022, there were a total of thirty concussions (n = 30). This translates to an incidence rate of 29 (19 to 39) concussions/1000 player hours and roughly *one concussion for every match played*. This was the second highest number of concussions recorded during the data collection period in these tournaments and has doubled since 2019.

The tournament with the highest concussion incidence rate was Gu16W, with 35 (19 to 52) concussions/1000 player hours (Table 8). These data are converted to 1 concussion event/match. There were no significant differences between the two tournaments.

**Figure 15:**
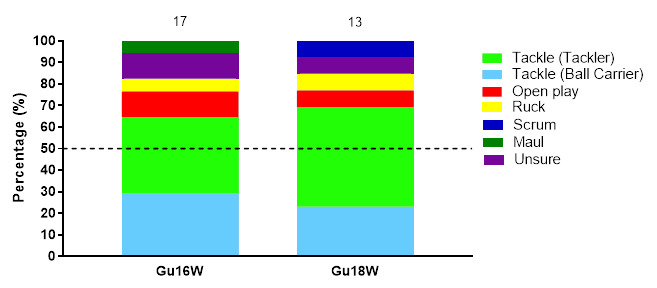
Proportion of concussions caused by the different injury events at the 2022 SARU Girls’ Youth Week Tournaments (n = 30 concussions; Gu16W = 17, Gu18W = 13). (The number above each bar represents the total number of concussions for that tournament). Missing 2022 data = 0 cases.

Tackling (40%, n = 12) contributed to the most concussions in 2022 (Figure 15), followed by *Being Tackled* (27%, n = 8). Figure 15 displays the proportion of concussions and their different injury-causing mechanisms across the two tournaments in 2022.

**Figure 16:**
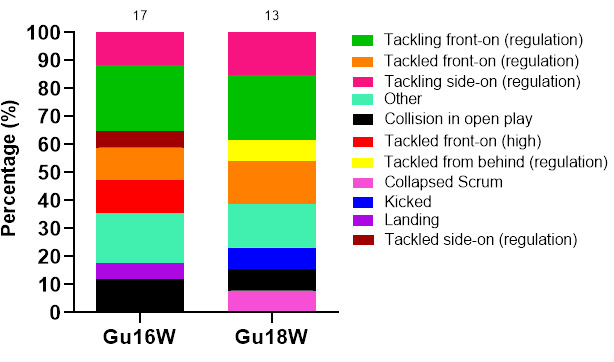
Proportion of concussions caused by the different injury mechanisms at the 2022 SARU Girls’ Youth Week Tournaments (The number above each bar represents the total number of concussions for that tournament). Missing 2022 data = 0 cases.

*Tackling front on (regulation)* accounted for 23% of all concussions, making it the most prominent singular event causing concussions for the combined tournament data (Figure 16). In Gu18W, *Tackling front on (regulation)* contributed to 3 of the 13 cases recorded in 2022. Furthermore, *Tackling front on (regulation)* in Gu16W, also contributed to 4 of the 17 cases (Figure 16).

**Figure 17:**
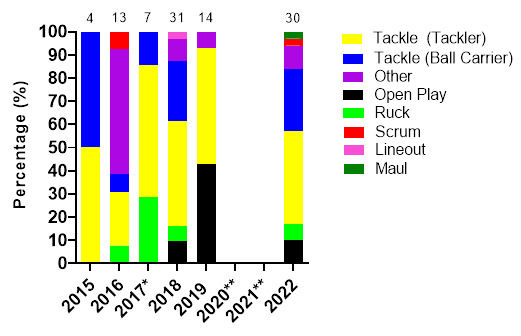
Proportion of concussions caused by the different injury events from 2015 to 2022 at the SARU Girls’ Youth Week Tournaments. (The number above each bar represents the total number of concussions for that year). *No Gu16W tournament was held in 2017. Missing 2022 data = 0 cases. **No Gu16W and Gu18W tournaments were held in 2020 and 2021 due to the COVID-19 pandemic.

Figure 17 displays the proportion of concussions caused by the different event mechanisms from 2015 to 2022. The *Tackle* event was the most prominent cause of concussions between 2015 and 2022, contributing to 64% of all concussions: 39% to the Tackler, 25% to the Ball Carrier. This could be due to poor tackle and ball carrying techniques; however, further video-analysis investigations are needed to verify this. Regardless, due to its high proportionate contributions towards all injuries and concussions, it is apparent, that *tackling*, and *ball-carrying* techniques need to be given more attention in the training and preparation of junior South African female rugby players.

The absolute number of concussions increased sizably since 2019, with 2022 being the second highest concussion number recorded at these tournaments.

**Figure 18:**
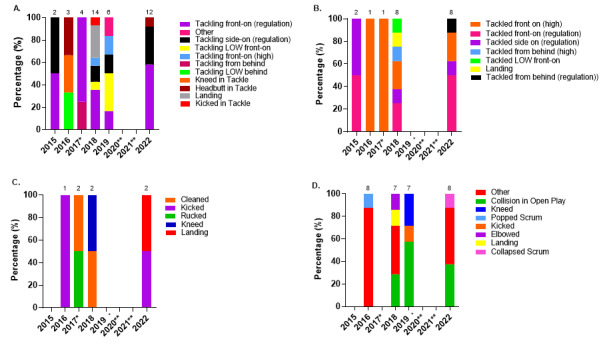
Proportionate breakdown of concussions caused by the various injury-causing mechanisms at the 2015 to 2022 SARU Girls’ Youth Week Tournaments. (The number above each bar represents the total number of concussions for that year in each graph category). **A.** Tackler-related concussion mechanisms **B.** Ball Carrier-related concussion mechanisms **C.** Ruck-related concussion mechanisms. **D.** Remaining concussion mechanisms. *No Gu16W tournament was held in 2017. Missing 2022 data = 0 cases. **No Gu16W and Gu18W tournaments were held in 2020 and 2021 due to the COVID-19 pandemic.

Figure 18 displays the proportionate breakdown of concussions resulting from the different injury-causing mechanisms over the six years studied.

Between 2015 and 2022, 42% of Tackler-related concussions (Figure 18A) were caused by *Tackling front-on (regulation)*, 35% of Ball Carrier-related concussions (Figure 18B) were caused by being *Tackled front-on (regulation)*, and 29% of Ruck-related concussions (Figure 18C) were caused by being *Kicked* in the breakdown contest and 29% by being *Cleaned out*.

**Figure 19:**
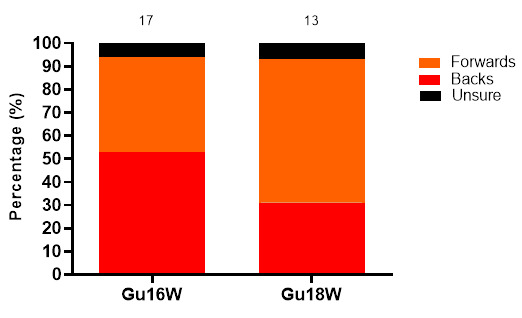
Proportionate breakdown of concussions for forwards and backs at the 2022 SARU Girls’ Youth Week Tournaments (the number above the bar represents the total number of concussions per category for that tournament). Missing 2022 data = 0 cases.

Concussions in 2022 were evenly distributed between forwards and backs. Additionally, at the Gu18W, 62% of concussions were sustained by forwards. (Figure 19).

**Figure 20:**
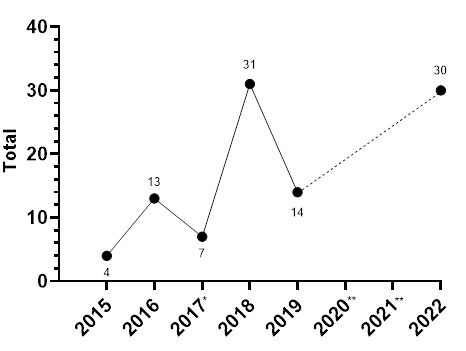
Total number of concussions per year at the SARU Girls’ Youth Week Tournaments from 2015 – 2022. (The number above each data point represents the total number of concussions for that year). *No Gu16W tournament was held in 2017. Missing 2022 data = 0 cases. **No Gu16W and Gu18W tournaments were held in 2020 and 2021 due to the COVID-19 pandemic.

**Figure 21:**
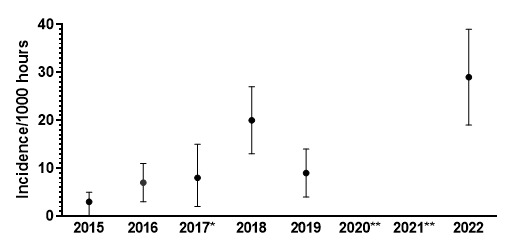
Concussion incidence rates and 95% confidence intervals/1000 player hours per year at the SARU Girls’ Youth Week Tournaments from 2015 – 2022. Missing 2022 data = 0 cases. *No Gu16W tournament was held in 2017. **No Gu16W and Gu18W tournaments were held in 2020 and 2021 due to the COVID-19 pandemic.

**Figure 22:**
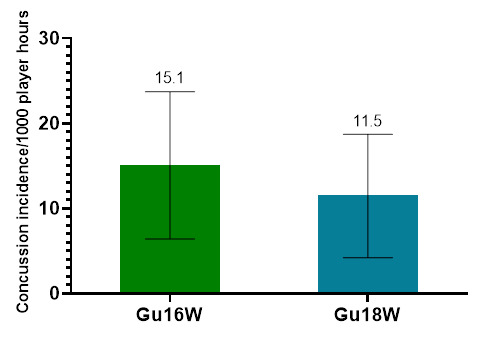
Concussion incidence rates and 95% confidence intervals/1000 player hours per SARU Girls’ Youth Week tournament from 2015 – 2022. No Gu16W tournament was held in 2017. No Gu16W and Gu18W tournaments were held in 2020 and 2021 due to the COVID-19 pandemic. The number above each bar represents the average concussion incidence/1000 player hours for the combined time-period of injury surveillance.

Figure 20 illustrates the total number of concussions from 2015 to 2022 for the SARU Girls’ Youth Week Tournaments. The corresponding concussion rate over the same period is shown in Figure 21. The total number of concussions increased sharply from 2019 to 2022, reaching a similar number to that recorded in 2018. A similar pattern was apparent in the incidence of concussion; although more matches were played and the incidence was slightly lower in 2018. This spike in 2022 could be attributed to the COVID-19 pandemic in 2020 and 2021. Players were unable to train, be coached, or compete, which may be a contributing cause to the again increased concussion numbers and rates seen in 2022. When combining all concussions over time per tournament (2015 – 2022), concussions and concussion rates tend to be lower at the Gu18W when compared to Gu16W (Figure 22). However, these differences are not significant.

**Figure f35-2078-516x-36-v36i1a18554:**
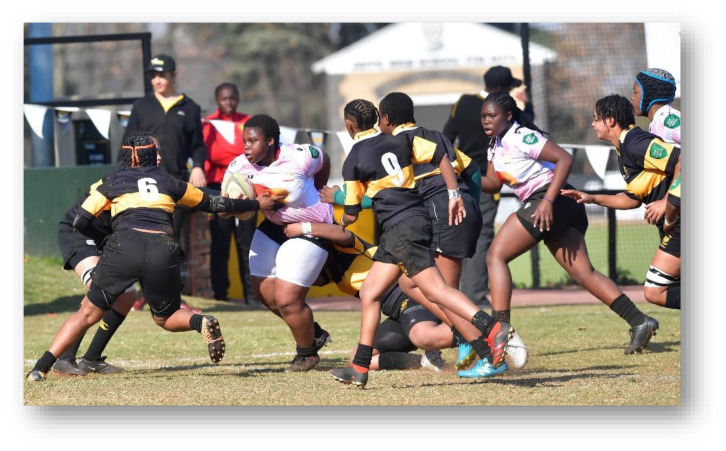

**Figure 23:**
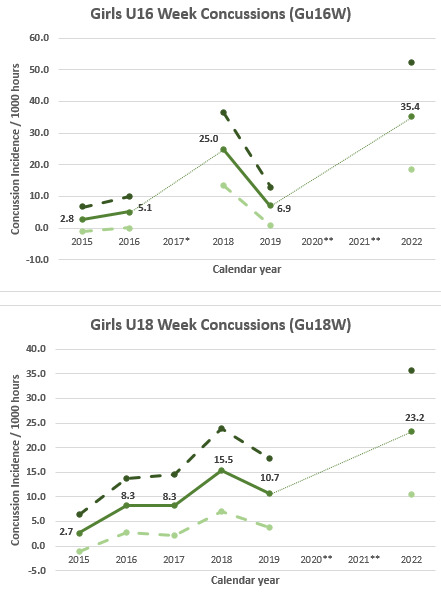
Concussion incidence and 95%CI for each SARU Girls’ Youth Week tournament, from 2015 – 2022. *No Gu16W tournament was held in 2017. **No Gu16W and Gu18W tournaments were held in 2020 and 2021 due to the COVID-19 pandemic.

The incidence of concussions during the Gu16W and Gu18W tournaments follows a similar trend to the injury rates. Concussion incidence rates increased gradually from around 2015 to 2018. From 2018 to 2019 there is a notable decrease in concussion incidence followed by a reversal and large increase in concussion incidence in 2022 (Figure 23). Concussion rates have increased 5-fold for Gu16W and 2-fold for Gu18W since 2019 (pre COVID-19) and the COVID-19 pandemic [IRR = 5.1 (2.2 to 11.2) for Gu16W and IRR = 2.1 (1.0 to 4.2) for Gu18W].

In Figure 23, there is no clear pattern of concussions for the Gu16W between 2015 and 2019. Concussions increase sharply from 2019 to 2022. There was no Gu16W tournament in 2017, 2020 and 2021, so the trendline could not be calculated with much accuracy and has therefore been excluded.

Similarly, in the Gu18W, the trendline could not be calculated accurately and therefore has also been excluded. In Gu18W, there was a sizable increase in concussions in 2018 with a recovery drop in concussions in 2019. In 2022 there was a prominent reversal and spike upwards in concussion incidence.

**Figure f36-2078-516x-36-v36i1a18554:**
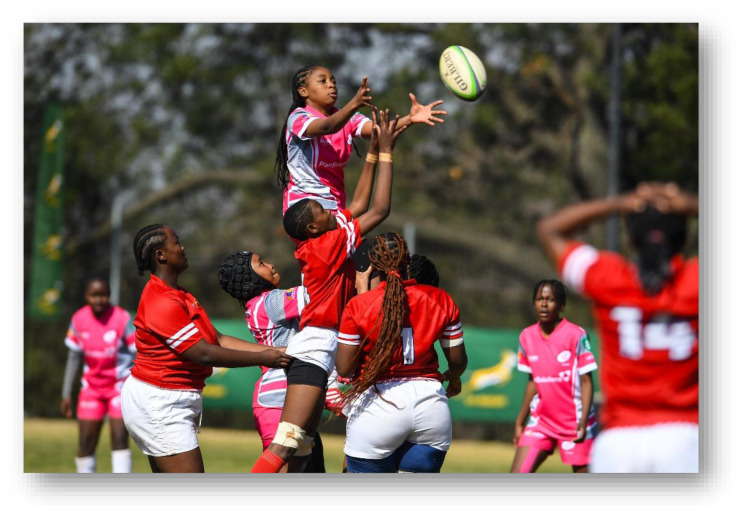

## Recommendations

1. The current format is that each team plays two matches per week, separated by a complete rest day. This format should remain in place.
2. It is important to increase the confidence and proficiency of young female rugby players in contact situations, as it contributes significantly to injuries and concussions. Developing the basic contact-safety skills, techniques, and required levels of proficiency through progressive and tailored training is crucial for junior South African female rugby players. This will help minimise concussions and other rugby-related injuries. It is important to prioritise such training to ensure that the players can safely tackle and carry the ball into contact.
3. Introduce the “Preparation for Contact” and “Contact Confident” programmes, freely available on the World Rugby education platform, to assist with the training and contact preparation of junior South African female rugby players.

**Figure f37-2078-516x-36-v36i1a18554:**
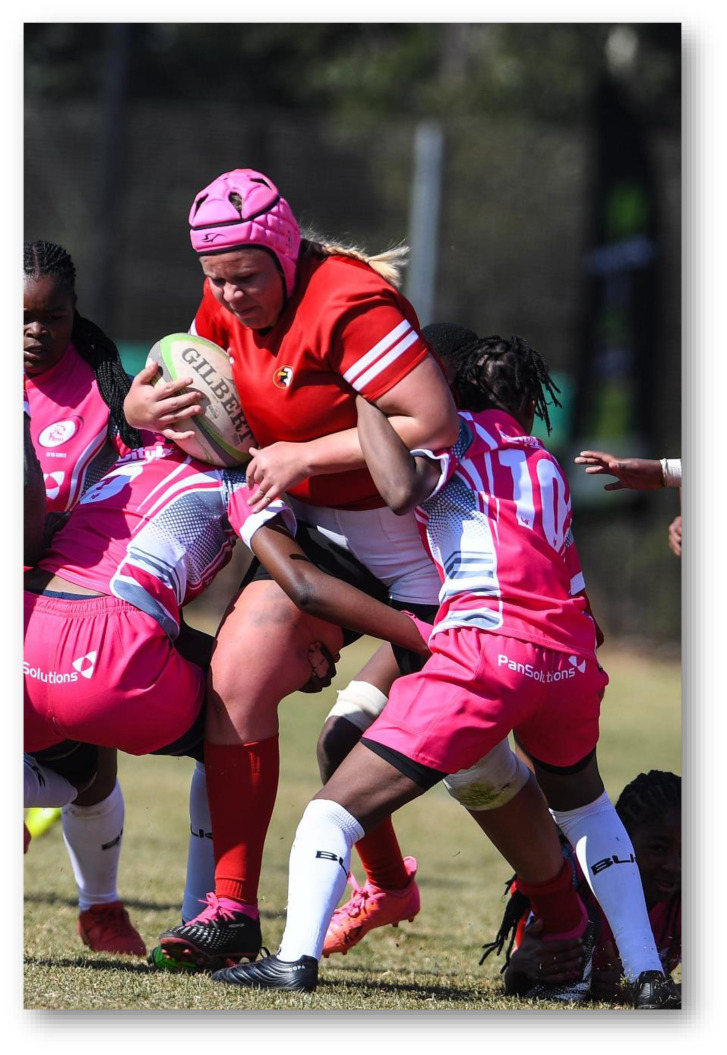

**Figure f38-2078-516x-36-v36i1a18554:**
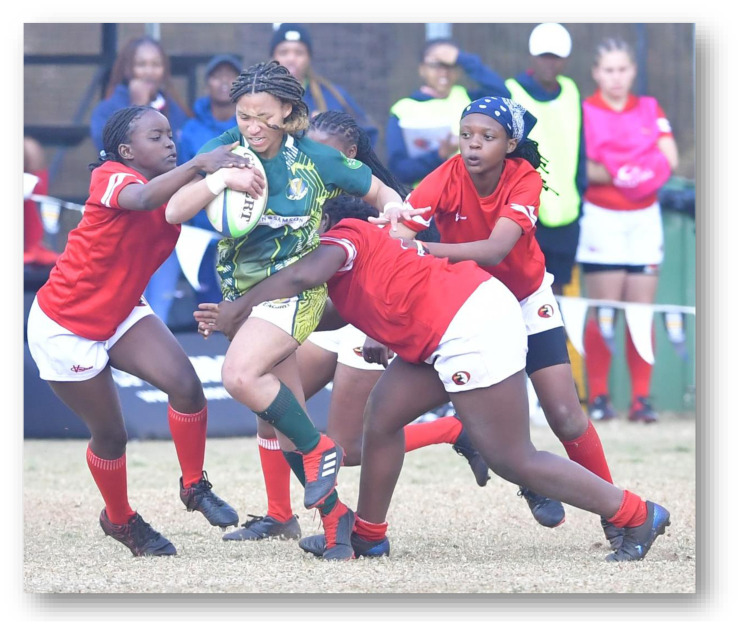

## Figures and Tables

**Table 1:** Number and injury incidence (95% CI)/1000 player hours of Medical Attention and Time-Loss injuries in the 2022 SARU Girls’ Youth Week tournaments.

	Medical Attention Injuries		Time-Loss Injuries	

	Number	Incidence	Number	Incidence
**Gu16W**	55	115 (84 – 145)	39	81 (56 – 107)
**Gu18W**	66	118 (89 – 146)	43	77 (54 – 100)

***Combined Total***	***121***	***116 (96 – 137)***	***82***	***79 (62 – 96)***

**Table 2:** Number of Medical Attention and Time-Loss injuries. Data expressed per match and per hour played in the 2022 SARU Girls’ Youth Week tournaments.

Tournament	Number of matches	Match duration (mins)	Medical Attention (injuries/match)	Time-Loss (injuries/match)	Medical Attention (injuries/hour)	Time-Loss (injuries/hour)
**Gu16W**	16	60	3.4	2.4	3.4	2.4
**Gu18W**	16	70	4.1	2.7	3.5	2.3

***Combined Tournament Average***	***16***	***65***	***3.8***	***2.6***	***3.5***	***2.4***

**Table 3:** Injury incidence (95% CI)/1000 player hours of Time-Loss injuries to the Tackler and Ball Carrier roles (within the Tackle), and Open Play, for the 2022 Girls’ SARU Youth Week tournaments.

Tournament	Tackler	Ball Carrier	Open Play
**Gu16W**	23 (9 – 37)	23 (9 – 37)	13 (3 – 23)
**Gu18W**	30 (16 – 45)	18 (7 – 29)	13 (3 – 22)

***Combined total***	***27 (17 – 37)***	***20 (12 – 29)***	***13 (6 – 19)***

**Table 4:** Injury incidence (95% CI)/1000 player hours of Time-Loss injuries at the 2022 SARU Girls’ Youth Week tournaments grouped as Central Nervous System (CNS), Joint/Ligament, and Muscle/Tendon injuries.

Tournament	CNS	Joint/Ligament	Muscle/Tendon
**Gu16W**	35 (19 – 52)	13 (3 – 23)	13 (3 – 23)
**Gu18W**	25 (12 – 38)	5 (0 – 11) *	16 (6 – 27)

***Combined Total***	***30 (19*** ** –** ***40)***	***9 (3 – 14)*** *	***14 (7 – 22)***

* Significantly lower than CNS injury types

**Table 5:** Proportion (%) and incidence (95% CI)/1000 player hours of Time-Loss injuries, grouped by body location, in the 2022 SARU Girls’ Youth Week tournaments.

	Proportion of injuries (%)	Incidence (95% CI)/1000 player hours
**Head and Neck**	48	38 (26 – 49) *
**Lower Body**	35	28 (18 – 38) *
**Upper Body**	13	11 (4 – 17)
**Trunk**	4	3 (0 – 6)

* Significantly higher than Trunk and Upper Body injury locations

**Table 6:** Injuries grouped according to the IOC recommended categories of Tissue and Pathology types for the 2022 SARU Girls’ Youth Week tournaments. Missing 2022 data for mean time loss = 13 cases.

Tissue	Injuries	Incidence	Mean time loss

*Pathology*	*n*	*Injuries per 1000 hours (95% CI)*	*Days (95% CI)*
**Muscle/Tendon**	15	14 (7 – 22)	5 (0 – 10)
*Muscle injury*	15	14 (7 – 22)	5 (0 – 10)
**Nervous**	31	30 (19 – 40)	28 (23 – 33)
*Brain/Spinal cord injury*	31	30 (19 – 40)	28 (23 – 33)
**Joint Sprain/Ligament tear**	9	9 (3 – 14)	12 (7 – 17)
**Bone**	5	5 (1 – 9)	14 (9 – 19)
*Fracture*	2	2 (0 – 5)	21
*Bone stress fracture*	1	1 (0 – 3)	-
*Bone contusion*	2	2 (0 – 5)	14
**Superficial tissue/skin**	10	10 (4 – 16)	5 (0 – 10)
*Contusion (superficial)*	10	10 (4 – 16)	5 (0 – 10)
**Other injury** *	12	12 (5 – 18)	12 (0 – 29)

***TOTAL***	**82**	***79 (62 – 96)***	**11 (6 – 16)**

Where n = 1, mean Time-Loss reflects the total Time-Loss days. Estimated severity for Time-Loss was used from data provided by the Tournament Medical Doctors at the venue when real-time severity was not able to be determined.

* All Other injuries were soft tissue injuries. Due to the nature of the data collection process, we were unable to delve deeper into determining a specific diagnosis.

**Table 7:** Number of injuries and injury rates (95% CI)/1000 exposure hours of players who started the match and those who came on as substitutes in the 2022 SARU Girls’ Youth Week tournaments. Missing 2022 data = 3 cases.

	Started match		Substitution	

	Number	Incidence	Number	Incidence
**Gu16W**	35	73 (49 – 97)	1	2 (0 – 6)
**Gu18W**	37	66 (45 – 87)	6	11 (2 – 19)
***Combined Total***	***72***	***69 (53 – 85)***	***7***	***7 (2 – 12)***

**Table 8:** Number and incidence of concussions (95% CI)/1000 player hours at the 2022 SARU Girls’ Youth Week tournaments.

Tournament	Number	Incidence	Number of matches per concussion event
**Gu16W**	17	35 (19 – 52)	1
**Gu18W**	13	23 (11 – 36)	1

***Combined Total***	***30***	***29 (19 – 39)***	***1***
